# Effects of Whole-Body Electromyostimulation on the Energy-Restriction-Induced Reduction of Muscle Mass During Intended Weight Loss

**DOI:** 10.3389/fphys.2019.01012

**Published:** 2019-08-12

**Authors:** Sebastian Willert, Anja Weissenfels, Matthias Kohl, Simon von Stengel, Michael Fröhlich, Heinz Kleinöder, Daniel Schöne, Marc Teschler, Wolfgang Kemmler

**Affiliations:** ^1^Institute of Medical Physics, Friedrich-Alexander University of Erlangen-Nürnberg, Erlangen, Germany; ^2^Department of Medical and Life Sciences, University of Furtwangen, Villingen-Schwenningen, Germany; ^3^Department of Sports Science, University of Kaiserslautern, Kaiserslautern, Germany; ^4^Institute of Training Science and Sport Informatics, German Sport University Cologne, Cologne, Germany; ^5^Institute of Rehabilitation Sciences, University of Witten/Herdecke, Witten, Germany

**Keywords:** electromyostimulation, energy restriction, weight loss, lean body mass, body composition, protein supplementation

## Abstract

**Purpose:** Overweight and obesity are an increasing problem worldwide. However, most studies that focus on weight reduction by energy restriction and/or aerobic exercise reported considerable loss of muscle mass as well. Increased protein intake and/or resistance exercise might inhibit this detrimental effect during a negative energy balance. Whole-body electromyostimulation (WB-EMS), a time effective, joint-friendly, and highly customizable training technology, showed similar hypertrophic effects compared with high-intensity resistance training. The aim of this study is to evaluate the effect of WB-EMS on body composition during negative energy balance with maintained/increased protein intake in overweight premenopausal women.

**Patients and Methods:** Ninety premenopausal, 25–50-year-old, overweight women were randomly assigned to three groups (*n* = 30 each). (1) Negative energy balance (−500 kcal/day) by energy restriction with compensatory protein intake (CG). (2) Negative energy balance (−500 kcal/day) by energy restriction (−250 kcal/day) and increased physical activity (−250 kcal/day) with increased protein intake (PA). (3) Negative energy balance (−500 kcal/day) due to energy restriction and increased physical activity with increased protein intake plus WB-EMS. The duration of the intervention was 16 weeks. Participants underwent restrictions in kcal per days and supplementation of protein (CG: 1.2 or PA/WB-EMS: 1.7 g/kg body mass/day) where needed. Bipolar WB-EMS was applied 1.5× week for 20 min (85 Hz; 350 μs; intermittent 6 s impulse, 4 s rest; rectangular). The primary study endpoint “lean body mass” (LBM) and secondary endpoint body fat mass (BFM) were assessed by bio-impedance analysis (BIA).

**Results:** LBM decreased in the CG and PA group (CG: −113 ± 1,872 g; PA: −391 ± 1,832 g) but increased in the WB-EMS group (387 ± 1,769 g). However, changes were not significant (*p* > 0.05). Comparing the groups by ANOVA, no significant differences were observed (*p* = 0.070). However, pairwise adjusted comparisons determined significant differences between WB-EMS and PA (*p* = 0.049). BFM decreased significantly (*p* < 0.001) in all groups (CG: −2,174 ± 4,331 g; PA: −3,743 ± 4,237 g; WB-EMS: −3,278 ± 4,023 g) without any significant difference between the groups (ANOVA: *p* = 0.131).

**Conclusion:** WB-EMS is an efficient, joint-friendly, and highly customizable training technology for maintaining muscle mass during energy restriction and can thus be considered as an alternative to more demanding resistance exercise protocols.

## Introduction

Overweight and obesity represent an increasing problem worldwide. According to newer statistics, more than 2.1 billion people worldwide are overweight or even obese (Smith and Smith, 2016). This development is of course primarily a health problem for the person affected: overweight and obesity are crucial risk factors for hypertension, fat metabolism disorders, diabetes mellitus type 2, cardiovascular diseases, and some types of cancer (Wilson et al., 2002; Villareal et al., 2005; Renehan et al., 2008). Further, the direct and indirect health costs for the treatment of secondary diseases, for example, and the higher risk of inability to work constitute an immense burden for the healthcare system (Lehnert et al., 2015). Smith and Smith (2016), for example, report several hundred billion dollars in costs caused by obesity in the United States.

Different strategies for generating a negative energy balance promise sustainable success in the fight against overweight and obesity. As has been proved, energy restriction leads to a loss of body mass. For health reasons, however, this reduction of body mass should focus on the loss of body fat, not muscle mass (Biesalski and Grimm, 2007). Creating a negative energy balance *via* pure restriction of calorie intake without looking at the macronutrients seems to result in a loss of lean body mass of up to one third of the total loss of body mass (Miller et al., 1997). However, there is evidence that supplementation with protein might reduce the loss of muscle mass during energy restriction (Pasiakos et al., 2013, 2015).

In addition to energy restriction alone, an increase in energy turnover through greater physical activity in daily life, particularly exercise training, also contributes to a negative energy balance. Endurance- and resistance-exercise training (RT) generate a sharp increase in energy consumption and thus contribute to a loss of body fat (Wilmore et al., 1999; Dietz et al., 2012). Apart from the acute effect of exercise, particularly intense bouts of resistance exercise affect energy demands through two further mechanisms. Adaptation and repair processes significantly increase energy demands up to 48–72 h post-exercise (Melby et al., 1993; Gillette et al., 1994; Paschalis et al., 2010). Furthermore, in contrast to endurance or mixed exercise protocols, hypertrophic effects induced by adequate resistance exercise increase the resting metabolic rate (RMR; Byrne and Wilmore, 2001; Hunter et al., 2008). Correspondingly, retention of lean body mass and consequently RMR in periods of weight reduction (Goran and Poehlman, 1992; Hunter et al., 2008) is of high relevance.

Thus, the combination of strength training and high protein intake during phases of energy restriction might be a reasonable intervention for decreasing body mass predominately or even exclusively by a reduction in body fat mass (Wycherley et al., 2010).

However, a lack of time might be the primary obstacle to start conventional exercise programs that focus on weight loss (Rütten et al., 2009). In addition, orthopedic problems due to overweight or simple aversion to heavy exercise might prevent a subject’s participation in exercise programs. Thus, time efficiency, joint friendliness, and high customization should be considered as a crucial aspect for effective interventions particularly for cohorts with low experience with or enthusiasm for physical exercise.

Whole-body electromyostimulation (WB-EMS), a novel time-saving, joint-friendly, and highly customizable training technology, might be a perfect candidate for cohorts with low time resources and/or low propensity for exercise (Kemmler et al., 2018). Recent studies have confirmed that WB-EMS has a comparable effect on hypertrophy, muscle strength, and fat loss as high intensity resistance training. Of interest, WB-EMS-induced reductions of body fat regularly increase the hypertrophic effect of this technology (Kemmler et al., 2016).

With regard to a personal-effective approach, the digital monitoring of training targets for increased physical activity in everyday life with the help of so-called activity trackers is of key importance. With the help of these trackers and the corresponding online training diary, a single trainer would thus have the opportunity to support his clients simultaneously without direct and prolonged contact with them and to check the activity specification. This combination of time-saving WB-EMS training, protein supplementation, and digital monitoring could be an effective, useful, and elegant solution in the setting of weight loss programs.

In summary, the aim of this study is to evaluate the effects of WB-EMS on body composition during energy restriction, increased physical activity in everyday life, and increased protein intake.

Our primary hypothesis is that WB-EMS, applied under energy restriction (ER), increased habitual physical activity in everyday life (PA) and high protein intake (PI), generates significantly more favorable changes on lean body mass than (1) isolated ER with moderate PI or (2) ER, high PI, and increased habitual physical activity in everyday life. Our secondary hypothesis is that (1) all three study arms generate significant reductions in absolute body fat mass (2) without significant group differences.

## Materials and Methods

### Trial Designs

The **W**eight-**R**eduction and **E**lectromyostimulation **P**lus **P**rotein (WREPP) project is a randomized, controlled, clinical intervention study (RCT) with three study arms in a parallel group design. The study was designed and conducted by the Institute of Medical Physics (IMP), Friedrich-Alexander University Erlangen-Nuremberg (FAU). The study follows the Helsinki Declaration “Ethical Principles for Medical Research Involving Human Subjects” and was approved by the Ethical Committee of the FAU (ethics application no. 19_16b). The project was fully registered under NCT03746977. All women participating were intensively informed about the study procedures. Written informed consent was obtained before each subject’s participation in the trial. We strictly followed the CONSORT 2010 guideline for randomized studies with a parallel group design (Moher et al., 2010).

#### Participants

**Figure 1:**
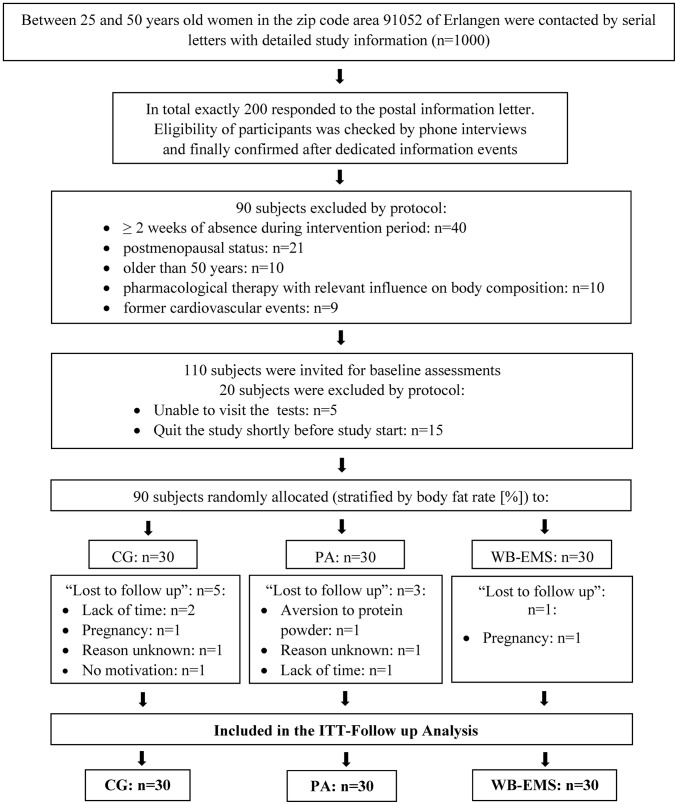
Flow diagram of the study intervention. *n*, numbers; CG, pure energy restriction with compensatory protein intake; PA, energy restriction and increased physical activity with increased protein intake; WB-EMS, energy restriction and increased physical activity with increased protein intake plus whole-body electromyostimulation; ITT, intention-to-treat.

Using citizen registers provided by the municipality, 1,000 women, 25–50 years old, living in the area of Erlangen-Nuremberg, Germany, were contacted by personal letters that already included the most important eligibility criteria. Two hundred women responded and were informed in more depth *via* a hotline and successive joint information events. Based on the eligibility criteria (1) 25–50 years old; (2) premenopausal status; (3) no diseases or pharmacological therapy with relevant influence on muscle mass and body fat (e.g., glucocorticoids >5 mg/day); (4) no pregnancy or acute breastfeeding period; (5) no conditions that exclude WB-EMS application (e.g., cardiac pacemaker); (6) no cardiovascular events (e.g., stroke, coronary infarction); and (7) no absence of more than 2 weeks during the study period, the eligible women (*n* = 110) were invited to determine obesity criteria by BIA (total body fat rate > 28%). Finally, 90 women fulfilled these criteria and agreed to participate in the study. Stratified according to body fat rate (%), participants were randomly allocated to three balanced intervention groups: (1) negative energy balance due to energy restriction with compensatory protein intake (CG: *n* = 30); (2) negative energy balance due to energy restriction and increased habitual physical activity in everyday life with increased protein intake (PA: *n* = 30); and (3) negative energy balance due to energy restriction and increased physical activity in everyday life with increased protein intake plus whole-body electromyostimulation (WB-EMS: *n* = 30). The flow chart of the study is shown in Figure 1, and the baseline characteristics of all the participants are illustrated in Table 1.

**Table 1:** Baseline characteristics of all groups (WB-EMS, PA, and CG; *n* = 30 per group).

Variable	WB-EMS MV ± SD	PA MV ± SD	CG MV ± SD	*p*
Age (years)^a^	38.4 ± 8.0	34.4 ± 8.3	35.3 ± 7.4	0.136
Body height (cm)^b^	167.6 ± 4.9	166.3 ± 5.6	167.5 ± 6.9	0.658
Body mass (kg)^c^	92.3 ± 17.6	84.8 ± 15.4	86.0 ± 17.8	0.195
BMI (kg/m^2^)	32.8 ± 6.8	30.5 ± 6.1	30.7 ± 6.2	0.327
Total body fat (%)^c^	42.0 ± 6.6	40.1 ± 7.2	39.2 ± 7.7	0.317
Energy consumption/d (kcal)^d^	2,299 ± 831	2,292 ± 555	2,178 ± 579	0.732
Protein intake/d (g)^d^	96 ± 33	88 ± 23	90 ± 22	0.506
Carbohydrate intake/d (g)^d^	240 ± 92	258 ± 59	231 ± 73	0.376
Total fat intake/d (g)^d^	94 ± 47	91 ± 30	89 ± 30	0.850
Hypertension (*n*)^a^	4	3	1	0.423
Smoking (*n*)^a^	4	3	4	0.896
Sport/week (min)^a^	74 ± 67	61 ± 68	91 ± 60	0.226
Steps/week (*n*)^e^	70,283 ± 21,105	65,302 ± 14,119	–	0.287
Physical condition (*n* of items)^f^	5 ± 1	5 ± 1	5 ± 1	0.901
Mental constitution (*n* of items)^f^	3 ± 2	3 ± 2	3 ± 1	0.683
Physical stress in daily life (*n* of items)^f^	3 ± 1	3 ± 1	3 ± 1	0.421

*WB-EMS, energy restriction and increased physical activity with increased protein intake plus whole-body electromyostimulation; PA, energy restriction and increased physical activity with increased protein intake; CG, pure energy restriction with compensatory protein intake; p, significance; d, day; kcal, kilocalories; g, gram; n, number; min, minutes*.

a *Assessed by baseline questionnaire*.

b *Measured by stadiometer*.

c *Measured by bio-impedance analysis (DSM-BIA, InBody 770, Seoul, South Korea)*.

d *Analyzed by Freiburger Nutrition Protocol (Nutri-Science, Hausach, Germany)*.

e *Measured by fitness tracker (Polar Loop 2, Polar Electro Oy, Kempele, Finland)*.

f Assessed by baseline questionnaire with a scale from 1 “very satisfied”/“very low” to 7 “very dissatisfied”/“very high.”

### Intervention

The interventions were applied for 16 weeks in all the groups (February 2018 until July 2018).

#### Group CG: Negative Energy Balance Due to Energy Restriction With Compensatory Protein Intake

Based on individual nutritional analysis (Freiburger Nutrition Protocol, Nutri-Science, Hausach, Germany) and nutrition consultations, participants had to achieve the 500 kcal/day energy reduction through a corresponding reduction of carbohydrates (i.e., ≈125 g/day). Multi-component protein powder (Hansepharm, Roth, Germany) was supplemented to ensure a total protein intake of 1.2 g/kg body mass/day. Women in the CG were asked to maintain their normal daily activity and exercise habits.

#### Group PA: Negative Energy Balance Due to Energy Restriction and Increased Habitual Physical Activity With Increased Protein Intake

As with the CG, participants had to reduce the amount of carbohydrates by 250 kcal/day (i.e., ≈62.5 g/day). Based on the dietary protocol, multi-component protein powder (Hansepharm, Roth, Germany) was supplemented to ensure a total protein intake of 1.7 g/kg body mass/day. In order to generate a negative energy balance of −500 kcal/day, the women were asked to increase their normal daily activity by 250 kcal/day by executing more daily steps at their habitual gait speed. For this purpose, a specific individual target of steps was calculated. Therefore, the mean value of pedestrian cadence (115.2 steps/min) under natural conditions (Tudor-Locke and Rowe, 2012) and the body mass of each participant were taken as a reference. Steps were monitored by a fitness tracker (Polar Loop 2, Polar Electro Oy, Kempele, Finland). The information, transmitted electronically to the study management, was checked regularly to ensure compliance with the training target. Nutritional and exercise guidelines should accordingly result in a reduction of energy balance of 500 kcal/day.

#### Group Whole-Body Electromyostimulation: Negative Energy Balance Due to Energy Restriction and Increased Habitual Physical Activity With Increased Protein Intake Plus Whole-Body Electromyostimulation

In addition to the treatment of the PA group, we applied a WB-EMS training 1.5 × 20 min/week (every Tuesday and every second Friday) using devices from miha bodytec (Gersthofen, Germany). Eight muscle groups (upper arms, chest, abdomen, latissimus, upper back, lower back, buttocks, thighs) were addressed by the WB-EMS (standard) application (85 Hz; 350 μs; intermittent; 6 s impulse phase, 4 s rest; rectangular; bipolar). Two sets of six to eight repetitions of easy movements with a small range of motion such as moderate squats, butterfly movements, and trunk flexions were carried out during the impulse phase. Sessions were supervised and guided by two certified instructors each responsible for two participants. In order to gradually guide the participants to the intended training load, the duration of the WB-EMS session was successively increased by 2 min from the first unit of 12 min until the full 20 min were reached in the fifth session. The intensity of training was based on the Borg CR 10 scale (Borg and Kaijser, 2006). The participants were instructed to realize a perceived exertion of between “5” (hard) and “7” (very hard). Trainers asked every 2 or 3 min, if they could raise the intensity of stimulation to adjust for the accustoming effect. The energy turnover of a single bout of WB-EMS (120 kcal/20 min session) (Kemmler et al., 2012) was considered in the calculation of the energy restriction (−225 kcal/day).

### Outcomes

Primary endpoint:

- Changes in lean body mass from baseline to 16-week follow-up.

Secondary endpoint:

- Changes of total body fat mass from baseline to 16-week follow-up.

### Assessments

All measurements were conducted at the IMP, FAU by dedicated researchers and research assistants. To ensure adequate standardization, the same test assessor using the same procedure carried out the dedicated baseline and follow-up measurement. All measurements were performed at similar times of the day (±1 h).

Calibrated devices were used for all assessments. Body height was measured barefoot using a stadiometer (Holtain Ltd., Crymych Dyfed, Great Britain). Body mass was assessed using bio-impedance technique (InBody 770, Seoul, South Korea), described in detail below. Body mass index (BMI) was calculated body mass (kg)/body height (m^2^).

All participants recorded their habitual diet as accurately as possible for 5 representative days (four weekdays and one weekend day) before and during the last week of the intervention. Using the corresponding evaluation software (Freiburger Nutrition Protocol, Nutri-Science, Hausach, Germany), the average volume of carbohydrate, fat, protein, and alcohol intake per day was calculated. Corresponding dietary changes and specification were discussed in detail with the participants by the principal investigator (SW). Based on the dietary analysis, the amount of carbohydrate intake was limited, by 56, 62.5, and 125 g/day, respectively, in order to generate a negative balance of 225 kcal/day (WB-EMS), 250 kcal/day (PA), and 500 kcal/day (CG). On the other hand, the intake of all the other macro-nutritional components was maintained. In parallel, protein supplements were provided to ensure a daily protein uptake of 1.2 g/kg body mass/day in the CG and 1.7 g/kg body mass/day in the PA and WB-EMS groups. We used a multicomponent protein powder (Hansepharm Power Eiweiß Plus, Roth, Germany), i.e., a mix of a whey, casein, soy, and egg protein blend with a high leucine (10.3%) and carnitine (1.7%) content. The same protocols and analysis were applied to check participants’ compliance with the dietary requirements, during the sixth and the last week of the intervention.

The calculation of the daily step target was based on the habitual walking volume of participants as determined by a fitness tracker (Polar Loop 2, Polar Electro Oy, Kempele, Finland). Trackers were worn for 2 weeks prior to the start of the intervention. Participants were instructed to maintain their normal daily activity and use representative days for the monitoring of their physical activity in everyday life. Considering the body mass of the participants, the daily walking volume in minutes required to consume 250 kcal of energy was calculated using an online calculator. Using the average number of steps per minute (115 steps/min) reported for “adult pedestrians” (Tudor-Locke and Rowe, 2012), the number of steps per day equivalent to an energy expenditure of 250 kcal/day was calculated. Of importance, all the tracked information were transmitted every fifth day to an online platform and submitted to the principal investigator (SW) who checked compliance of the participants and, if necessary, asked participants to increase (or decrease) their daily step number in order to properly comply with the physical activity protocol.

Direct segmental, multi-frequency BIA-technique (Inbody 770, Seoul, South Korea) was applied to evaluate body composition including the primary (LBM) and secondary (BFM) study outcome. The device separately measures trunk, arms, and legs using a tetrapolar eight-point tactile electrode system by means of six different frequencies (1, 5, 50, 250, 500, and 1,000 kHz). Among the participants of the present study, intra class correlation (ICC, test-retest) of LBM as assessed by the Inbody 770 was 0.90, and ICCs for percent body fat was comparably high (0.88).

#### Sample Size

Based on a 5% difference in lean body mass between the WB-EMS group and the PA group (MV, SD: 6.5%), 27 persons/group had to be included to generate at least a power of 80% based on an *α* = 0.05 (*t* test based sample size calculation). Anticipating a “loss to follow-up” rate of 10%, we included 30 women/group in order to perform an adjuvant per protocol analysis.

#### Randomization

Stratified for body fat rate (%) and consistently supervised by the principal investigator (SW), the participants drew lots and allocated themselves to the three conditions. Lots were put in opaque plastic shells and were drawn by the participants from the same bowl in the order of their appearance. Of importance, neither participants nor researchers knew the allocation beforehand. After the assignment into three equal groups (*n* = 30/each), each woman was informed in detail by the principle investigator (SW) about dos and don’ts.

#### Blinding

Due to the study design, we were unable to properly blind participants with respect to their allocation. However, researchers conducting the tests or analysis were not aware of the participants’ group status and were not allowed to ask, either.

### Statistical Analyses

Analyses were carried out using the statistical software R (R Development Core Team, 2018) and SPSS 25 (SPSS Inc., Chicago, IL, USA). Data were reported using mean values (MV) and standard deviation (SD). We applied an intention to treat analysis with multiple imputation for missing values, using Amelia II, which is implemented in the R package “Amelia” (Honaker et al., 2011). Following the recommendations of Honaker et al. (2011), the complete data set was used for imputation. In order to obtain stable results, 100 imputed data sets were generated. The “overimpute” plot was used to control the imputations. The plots showed that the imputations for the variables to the primary and secondary endpoints had worked very well. Statistical (Shapiro-Wilk test) and graphical (QQ plots) tests indicated a normal distribution for the measured endpoints. To evaluate intra-group changes dependent *t* test were applied. Inter-group differences were analyzed using ANOVA. In case of relevant differences (*p* < 0.150), pairwise differences between the groups were checked using independent *t* tests, adjusted for multiple testing according to the method of Barnard and Rubin (Barnard and Rubin, 1999). Nominal scaled data, reported in Table 1, were analyzed by *χ*^2^ tests. All tests were two tailed, and statistical significance was accepted at *p* < 0.05.

## Results

Baseline characteristics are given in Table 1. As stated, no significant between-group differences were determined (Table 1).

Nine participants (WB-EMS: *n* = 1 vs. PA: *n* = 3 vs. CG: *n* = 5) were lost to follow-up. Reasons given for withdrawal were (1) lack of motivation (*n* = 2); (2) pregnancy (*n* = 2); (3) lack of time (*n* = 2); and (4) aversion to protein powder (*n* = 1). Two women apparently lost interest and did not give reasons for their withdrawal.

Due to the possibility to catch up missed sessions, the attendance rate in the WB-EMS group was 100% (i.e., 24 sessions). None of the participants reported any adverse effects or complaints caused by the training. Further, based on our logs, compliance with the prescribed protein (powder) dose was high. Personal interviews with the participants (SW) showed a strict compliance with the protein supplementation protocol and confirmed this finding. However, slight decreases in dietary protein intake were observed, thus none of the study groups fully realized the intended total protein intake (WB-EMS: 1.67 ± 0.11 vs. PA: 1.62 ± 0.12 vs. CG: 1.16 ± 0.13 g/kg body mass/day).

Five-day dietary intake protocols conducted by all the participants indicated that average energy reduction by carbohydrate restriction, i.e., 500 kcal/day in the CG, and 225 and 250 kcal/day for the WB-EMS and PA group, respectively, was significantly exceeded (*p* ≤ 0.007) in all the groups. However, considerable differences among the participants of all three groups were observed. Energy reduction reported by participants in the CG averaged 678 ± 505 kcal/day (range: 110 to −1,433 kcal); corresponding energy reduction was 528 ± 477 kcal/day (range: 301 to −1,261 kcal/day) in the PA and 462 ± 399 kcal (range: 97 to −1,177 kcal/day) in the WB-EMS group. We further observed that the WB-EMS (198 ± 40 kcal/day) and the PA (180 ± 38 kcal/day) groups failed to generate the intended physical activity level corresponding to 225/250 kcal/day. Thus, overall reductions of net energy balance were 678 ± 505 kcal/day in the CG, 708 ± 519 kcal/day in the PA, and, considering the energy cost of the WB-EMS application, 660 ± 501 kcal in the WB-EMS group. Using ANOVA, significant between-group differences were not observed for the latter parameter (*p* = 0.660).

The primary study endpoint “lean body mass” (Table 2) decreased in the CG (−0.2 ± 2.9%) and PA groups (−0.8 ± 1.8%) but increased in the WB-EMS group (0.5 ± 2.5%). However, none of these intra-group changes (*p* ≥ 0.11) were statistically significant. In parallel, no significant differences between the three groups were determined by ANOVA (*p* = 0.070) (Table 2). However, pairwise comparisons adjusted for multiple testing indicated a borderline significant difference between WB-EMS and PA (*p* = 0.049), while other comparisons, i.e., CG vs. PA (*p* = 0.424) or CG vs. WB-EMS (*p* = 0.390) were far from being significant (Table 2). Thus, we confirm our primary hypothesis (2) that WB-EMS generated significantly more favorable changes on lean body mass than a similar intervention, however without EMS-application (i.e., PA group). However, we have to reject our primary hypothesis (1) that WB-EMS, PA (250 kcal/day), energy restriction (225 kcal/day), and high protein intake (1.7 g/kg body mass/day) generated significantly more favorable changes on LBM than a control group with isolated energy restriction (500 kcal/day) and moderate protein intake (1.2 g/kg/body mass/day).

**Table 2:** Baseline value, changes (i.e., pre-post difference per group) of primary and secondary study endpoints, and significance levels.

	WB-EMS MV ± SD	PA MV ± SD	CG MV ± SD	*p*
**Lean body mass (g)**				
Baseline	52,707 ± 6,319	49,883 ± 4,799	51,250 ± 6,063	0.172
Difference	387 ± 1,769	−391 ± 1,832	−113 ± 1,872	0.070
**Absolute body fat mass (g)**				
Baseline	39,580 ± 13,428	34,953 ± 12,309	34,733 ± 13,878	0.282
Difference	−3,278 ± 4,023^***^	−3,743 ± 4,237^***^	−2,174 ± 4,331^***^	0.131

*WB-EMS, energy restriction and increased physical activity with increased protein intake plus whole-body electromyostimulation; PA, energy restriction and increased physical activity with increased protein intake; CG, pure energy restriction with compensatory protein intake; p, significance; MV, mean value; SD, standard deviation*.

*** *p < 0.001*.

Total body fat mass decreased significantly (*p* < 0.001) in all the groups (CG: −6.3 ± 7.8%; PA: −10.7 ± 8.7%; WB-EMS: −8.3 ± 7.4%) without significant group differences (*p* = 0.131) as determined by ANOVA (Table 2). After adjusting for multiple testing, pairwise comparisons did not result in significant differences between the groups (CG vs. PA: *p* = 0.142; CG vs. WB-EMS: *p* = 0.331; PA vs. WB-EMS: *p* = 0.578). Thus, we fully confirm our secondary hypothesis that (1) all three groups generated significant reductions in total body fat mass (2) without significant group differences.

Changes in parameters that might confound our results were not detected. None of the participants reported any new disease or change of relevant medication or smoking habits during the study period. Physical activity and exercise outside the study increased in all the groups (CG: *p* = 0.034 vs. PA: *p* = 0.257 vs. WB-EMS: *p* = 0.076), however, without significant between-group differences (*p* = 0.800).

## Discussion

The general purpose of the study was to evaluate the effect of WB-EMS on body composition, under specific consideration of LBM changes, during negative energy balance, but increased protein intake in overweight 25- to 50-year-old premenopausal women. In summary, all of the interventions favorably affect LBM (<10%). Compared with the 25–30% LBM contribution to total weight loss reported by others (Ballor et al., 1988; Miller et al., 1997; Weinheimer et al., 2010), WB-EMS in combination with high protein intake seems to be the most suitable method for maintaining muscle mass during intended, mild decreases of net energy balance by caloric restriction and/or increased physical activity. Addressing changes in total body fat mass, all the groups significantly reduced fat mass during the 16-week intervention period (−6.3 ± 7.8 to −10.7 ± 8.7%).

This study is the first trial to determine the effect of WB-EMS on body composition during intended and structured negative energy balance. In summary, the present study provided evidence for introducing WB-EMS in treating overweight and obesity. Considering the hypertrophic effects of WB-EMS, a recent study demonstrates positive effects in the range of a high-intensity resistance training (HIT-RT). Applying both types of exercise for 16 weeks in untrained men, 30–50 years old (*n* = 46), Kemmler et al. (2016) reported comparable (*p* = 0.395), highly significant increases in LBM in the WB-EMS (0.63 ± 0.77 kg) and HIT (0.86 ± 0.97 kg) study arm of its randomized controlled trial (RCT). Of importance for the present topic, most WB-EMS studies reported reductions in fat mass that considerably exceeded the increases in muscle mass, resulting in a net weight loss (Kemmler et al., 2018). However, in contrast to HIT-RT, WB-EMS is a joint friendly and safe training method (Kemmler et al., 2016) feasible for all people unable or unwilling to join conventional (resistance) exercise programs. Due to the lack of other WB-EMS studies in this field, however, we now discuss results of RT trials to allow the reader to consider the relevance of our results.

Reviewing the literature, several studies indicated that isolated energy restriction protocols reduce body fat mass and LBM (Ballor et al., 1988; Miller et al., 1997; Weinheimer et al., 2010). Applying a hypocaloric diet in 40 obese people, Ballor et al. (1988) reported that 25% of total body mass loss can be attributed to LBM reductions. This result was confirmed by the systematic review of Weinheimer et al. (2010) that included trials examining overweight postmenopausal women under caloric restrictions of 9–52 weeks and calculated an average decrease in fat-free mass of about 25% of weight loss. In the “diet only” study arm of their meta-analysis (493 studies) with overweight adults, 2–90 weeks long, Miller et al. (1997) reported a body-mass reduction that constituted two thirds fat and one third fat-free mass.

Revisiting promising methods to stop or attenuate the loss of LBM during energy restriction, the current state of research suggests resistance exercise training. In their systematic review and meta-analysis, Sardeli et al. (2018) observed that RT, particularly when applied in multiple sets over 12–24 weeks, stops the decline of LBM in obese elderly people during a phase of caloric restriction (472 and 800 kcal/day; Sardeli et al., 2018). Hunter et al. (2008) investigated the effect of RT on fat-free mass in premenopausal overweight women during a diet-induced (800 kcal/day, 21 weeks) weight loss of approximately 12 kg. Compared with participants without exercise training (47.9 ± 4.7 to 46.4 ± 5.1 kg) or aerobic training (45.4 ± 4.2 to 44.4 ± 4.1 kg), RT maintained fat-free mass (46.9 ± 5.2 to 47.2 ± 5.0 kg) to a similar degree as in the present study (Hunter et al., 2008). Monitoring 40 obese premenopausal females in an eight-week weight-loss study, Ballor et al. (1988) draw a similar conclusion. Following a daily deficit of 1,000 kcal and supplementing protein to ensure a protein intake of ≥1.0 g/kg body mass/day, the exercise group conducting RT 3 days per week preserved and even significantly increased LBM compared with the “diet only” group (0.43 ± 0.26 vs. −0.91 ± 0.28 kg; *p* < 0.05).

Another possibility to protect LBM during a phase of negative energy balance might be a higher daily protein intake. In their meta-analysis of 27 RCTs, Stonehouse et al. (2016) compared the effects of dairy products (including whey protein) on body composition during energy restriction in 18–50-year-old predominately overweight to obese adults (*n* = 1,278) (Stonehouse et al., 2016). The daily energy restriction of the trials was usually 500 kcal or more for the average of 16 weeks. In summary, intervention groups with increased protein intake lost (non-significantly) less LBM than control groups (−0.12 ± 1.57 vs. −0.56 ± 1.54 kg). Of importance, the authors observed the most favorable effects on LBM maintenance using a protein intake of >1.2 g/kg body mass/day and additional RT. Similar findings were provided by another meta-analysis or meta-regression (Krieger et al., 2006; Wycherley et al., 2012). The comparison of 24 RCTs by Wycherley et al. (2012) with adults (mean study duration 12.1 ± 9.3 weeks) under energy restriction (exact amount not specified) demonstrated that diets with high protein intake (1.25 ± 0.17 g/kg body mass/day) resulted in significantly lower reductions in fat-free mass than diets with standard protein consumption (0.72 ± 0.09 g/kg/day). However, differences were significant for interventions longer than 12 weeks only. Krieger et al. (2006) reported that the degree of FFM retention during energy-restricted weight loss tended to increase with each successive quartile of dietary protein intake and that protein intake of 1.05 g/kg body mass/day or more might improve FFM retention. A 12-week RCT with 46, 28–80-year-old overweight and obese women on energy deficit (750 kcal/day) compared the effect of normal versus higher (0.92 vs. 1.52 g/kg body mass/day) protein intake (Leidy et al., 2007). In summary, the high protein group lost less LBM than the lower protein group (−1.5 ± 0.3 vs. −2.8 ± 0.5 kg; *p* < 0.05). This finding confirmed the results of an earlier trial (Farnsworth et al., 2003) with 43 overweight and obese women, 20–65 years old, on a 12-week energy restrictive diet (≈−500 kcal/day). Again, participants with higher protein intake (1.4 g/kg body mass/day) lost less LBM (−0.1 ± 0.3 kg vs. −1.5 ± 0.3 kg, *p* = 0.02) than their peers’ standard protein consumption (0.8 g/kg body mass/day). Summing up the results in his systematic review, Pasiakos et al. (2013) recommend a daily protein intake twice (1.6 g/kg body mass) or three times (2.4 g/kg body mass) as high as the usual standard recommendation (0.8 g/kg body mass) to preserve lean body mass.

Although a recent meta-analysis (Stonehouse et al., 2016) did not fully confirm this strategy, a combination of strength training and increased protein intake during energy restriction might promise additive effects on LBM retention. Closest to our study, Layman et al. (2005) provided a high protein (1.6 g/kg body mass/day) reduced carbohydrate diet (400–500 kcal/day) combined with RT twice a week for 16 weeks for 48 overweighed women, 40–56 years old. In summary, this protocol resulted in more favorable changes in LBM (−0.9%) and fat mass (−22%) than a low protein (0.8 g/kg body mass/day) high carbohydrate diet (−5.4 and −12%) or an isolated high protein diet (−4.0 and 18%). The authors (Layman et al., 2005) applied a single set RT to fatigue of 30 min, a protocol with similar effects on body composition compared with WB-EMS (Kemmler et al., 2016).

Apart from effectiveness, adherence to the exercise protocol is a crucial aspect of weight loss (Acharya et al., 2009). In summary, a recent systematic review on WB-EMS reported low dropout (<10%) and high attendance (>90%) rates without any injuries (Kemmler et al., 2018). Even excluding studies with a high dropout (i.e., >30%), in their systematic review addressing early postmenopausal women, Asikainen et al. (2004) reported less favorable findings for RT, aerobic, or mixed protocols (Asikainen et al., 2004).

Summing up, we conclude that WB-EMS combined with high protein intake is an effective and feasible option for maintaining LBM under negative net energy balance and thus preventing a decrease of RMR with negative consequences on further weight management. However, some features and study limitations might decrease the evidence of the present trial or at least aggravate its proper interpretation. (1) One may argue that the study design with three groups and a mix of energy restriction, protein supplementation with different dose, physical activity, and WB-EMS might be too sophisticated and complex for a single study. Retrospectively, we partially agree with this criticism; however, the aim of the study was to determine the specific effect of WB-EMS on LBM retention applying a “state of the art” weight loss protocol^1^. As discussed in detail above, this includes adequate protein supplementation, which was provided in all the groups, albeit in different doses (1.7 vs. 1.2 g/kg/body mass/day). The reason for the latter strategy was the increased demand for protein due to RT- (or WB-EMS-) induced negative muscle protein balance (Phillips et al., 1997). (2) Considering the rather modest decreases in fat mass (−6.3 ± 7.8 to −10.7 ± 8.7%) in this cohort of overweight to obese women, it is unlikely that net energy deficiency truly fell within the range of about 650–700 kcal/day (i.e., ≈75.000 kcal/16 weeks). While physical activity was tracked by calibrated, valid devices and can be thus considered as a reliable outcome (Bunn et al., 2019), results on energy restriction as reported by the participants were dubious. Although we strictly emphasized a practicable protocol that focused on carbohydrate reduction only, extensively discussed the dietary protocol with the participants and contacted participants every second week to check compliance with the dietary recommendations, the majority of participants obviously “over-reported” their caloric restriction. (3) We applied multi-segmental multi-frequency bio-impedance analysis (BIA) to determine body composition of our cohort. However, the more common way of assessing body composition in research is dual-energy X-ray absorptiometry (DXA) – considered as the gold standard in body composition assessment. Recent comparisons, however, report that BIA appears to be an adequate alternative - not only because of its easier handling in the clinical setting and the lack of x-rays (Fosbol and Zerahn, 2015; Gomez-Arbelaez et al., 2017). Fosbol and Zerahn (2015) and Gomez-Arbelaez et al. (2017) compared different methods of measurement for body composition and concluded that there is no general recommendation for any type of measurement, since every single method has its advantages and disadvantages. Our own research has confirmed this conclusion. In studies with different cohorts (Von Stengel et al., 2013; Kemmler et al., 2017), we observed a high agreement for lean and fat mass between the Inbody 770 used in this trial and a Hologic 4500a DXA Scanner with a narrow limit of agreement. Even more important, the reliability of the BIA device to determine muscle mass determined by a test-retest approach with 25 participants resulted in high intra class correlation (ICC) of 0.86–91 (95% CI: 84–94) for LBM (Kemmler et al., 2017). (4) Although physical activity is frequently listed as a component of weight reductions programs (PA & WB-EMS), its transfer to everyday practice is problematical. With respect to our aim of 250/225 kcal energy consumption by physical activity in everyday life, the increase of daily walking ranged from 6,000 to 10,000 additional steps. Since all the women are employed and the predominant majority of them has to manage their families including younger children, it is understandable that most of them failed to realize their daily step specification. The latter aspect might further hinder the generality of our result. Additionally, the wide range of overweight and obesity (28–55% body fat rate) within our study might limit the proper transfer to dedicated premenopausal cohorts. Thus, more dedicated eligibility criteria for the present study might have generated more meaningful and transferable results.

In summary, the combination of WB-EMS and higher protein intake is an effective tool for favorably affecting body composition in overweight premenopausal women following a moderate energy deficit. Considering the time efficiency, joint friendliness and high degree of customization of this novel training technology, WB-EMS might be a feasible alternative to RT at least in people unmotivated or unable to join demanding resistance exercise protocols during their weight loss programs. However, further WB-EMS studies are needed to overcome the limitations of the present study and to check transferability on other cohorts.

## Data Availability

The raw data supporting the conclusions of this manuscript will be made available by the authors, without undue reservation, to any qualified researcher.

## Ethics Statement

This study was carried out in accordance with the recommendations of “Declaration of Helsinki”, with written informed consent from all subjects. The protocol was approved by the “Ethics Commission of the Friedrich-Alexander University Erlangen-Nürnberg” (number: 19_16b).

## Author Contributions

SW, AW, SS, DS, MT, and WK designed the study, completed the data analysis on each location and/or interpretation, and drafted the manuscript. MF and HK contributed to study conception and design and contributed to revise the manuscript. MK performed the statistical analysis of the data. SW accepts responsibility for the integrity of the data sampling, analysis, and interpretation.

### Conflict of Interest Statement

The authors declare that the research was conducted in the absence of any commercial or financial relationships that could be construed as a potential conflict of interest.

The handling editor declared a past co-authorship with several of the authors WK and MK.

## Footnotes

^1^Considering that LBM reduction in all of the study groups averaged below 10% of total weight loss we obviously realized this aim.…
